# BODY MASS INDEX AND COGNITIVE FUNCTION DECLINE

**DOI:** 10.1093/geroni/igz038.3006

**Published:** 2019-11-08

**Authors:** Yingxiao Hua, Yingxiao Hua, Dexia Kong, XinQi Dong

**Affiliations:** 1 Rutgers Institute for Health, Health Care Policy and Aging, New Brunswick, New Jersey, United States; 2 Rutgers University, The State University of New Jersey, New Brunswick, New Jersey, United States

## Abstract

Body composition has been proposed as an important modifiable risk factor of cognitive decline in multiple epidemiological studies. However, the relationship between body mass index (BMI) and cognitive function remains controversial and conflicting in diverse populations. This study aims to investigate the association between BMI and cognitive decline in U.S. Chinese older adults. Classifications of BMI are based on Asian criteria recommended by WHO (underweight: BMI<18.5, normal weight: 18.5≤BMI<23, overweight: 23≤bmi<27.5, obesity: bmi≥27.5). Logistic regression models were conducted. Compared with normal-weight participants, underweight participants were more likely to experience decline in episodic memory (OR=1.68, p=0.002) and work memory (OR=1.38, p=0.05). Being overweight and obesity were not associated with cognitive function decline. The findings indicate that underweight could potentially be a risk factor of cognitive function decline among U.S. Chinese older adults. Perspective studies may further investigate the association between weight loss and cognitive decline for the development of prevention strategies.

